# Epidemiological trends, antifungal drug susceptibility and *SQLE* point mutations in etiologic species of human dermatophytosis in Al-Diwaneyah, Iraq

**DOI:** 10.1038/s41598-024-63425-w

**Published:** 2024-06-03

**Authors:** Hussein R. Mahmood, Masoomeh Shams-Ghahfarokhi, Zahra Salehi, Mehdi Razzaghi-Abyaneh

**Affiliations:** 1 Department of Mycology, Faculty of Medical Sciences, Tarbiat Modares University, Tehran, 14115-331 Iran; 2 Department of Pathological Analysis, Faculty of Sciences, University of Al-Qadisiyah, Al-Qadisiyah, Iraq; 3 Department of Mycology, Pasteur Institute of Iran, Tehran, 1316943551 Iran

**Keywords:** Dermatophytes, Antifungal susceptibility, Dermatophytosis, Terbinafine resistance, *SQLE* mutation, Iraq, Biological techniques, Microbiology

## Abstract

Dermatophytes show a wide geographic distribution and are the main causative agents of skin fungal infections in many regions of the world. Recently, their resistance to antifungal drugs has led to an obstacle to effective treatment. To address the lack of dermatophytosis data in Iraq, this study was designed to investigate the distribution and prevalence of dermatophytes in the human population and single point mutations in squalene epoxidase gene (*SQLE*) of terbinafine resistant isolates. The identification of 102 dermatophytes isolated from clinical human dermatophytosis was performed through morphological and microscopic characteristics followed by molecular analysis based on ITS and *TEF-1α* sequencing. Phylogeny was achieved through RAxML analysis. CLSI M38-A2 protocol was used to assess antifungal susceptibility of the isolates to four major antifungal drugs. Additionally, the presence of point mutations in *SQLE* gene, which are responsible for terbinafine resistance was investigated. Tinea corporis was the most prevalent clinical manifestation accounting for 37.24% of examined cases of dermatophytosis. Based on ITS*, T. indotineae* (50.98%),* T. mentagrophytes* (19.61%)*,* and* M. canis* (29.41%) was identified as an etiologic species. *T. indotineae* and *T. mentagrophytes* strains were identified as *T. interdigitale* based on *TEF-1α*. Terbinafine showed the highest efficacy among the tested antifungal drugs. *T. indotineae* and *T. mentagrophytes* showed the highest resistance to antifungal drugs with MICs of 2–4 and 4 μg/mL, while *M. canis* was the most susceptible species. Three of *T. indotineae* isolates showed mutations in *SQLE* gene Phe^397^Leu substitution. A non-previously described point mutation, Phe^311^Leu was identified in *T. indotineae* and mutations Lys^276^Asn, Phe^397^Leu and Leu^419^Phe were diagnosed in *T. mentagrophytes* XVII. The results of mutation analysis showed that Phe^397^Leu was a destabilizing mutation; protein stability has decreased with variations in pH, and point mutations affected the interatomic interaction, resulting in bond disruption. These results could help to control the progression of disease effectively and make decisions regarding the selection of appropriate drugs for dermatophyte infections.

## Introduction

Dermatophytosis is a superficial infection of nails, hair, and skin in humans and animals caused by dermatophytes^1^. Based on the latest classification, dermatophytes can be classified into seven distinct genera, including *Trichophyton, Epidermophyton, Nannizzia, Paraphyton, Lophophyton, Microsporum*, and *Arthroderma*^2^. Among these genera, recent epidemiological studies have shown a trend towards an increased occurrence of *T. mentagrophytes/T. interdigitale* species complex surprisingly turned out to be the most common dermatophyte with a prevalence of up to 75.9 to 77.5%^3^.

Since the taxonomy of the *T. mentagrophytes/T. interdigitale* species complex is still in dispute, with three potential names being available for the Indian outbreak, an in-depth taxonomic study was undertaken combining molecular, morphological, and physiological characteristics as evidence of classification^4,5^. *T. mentagrophytes*/*T. interdigitale* species complex phenotypic characters are not diagnostic but statistically showed significant differences between the molecular siblings. These properties may be drivers of separate evolutionary trends^4^. Also, the origin of the outbreak is ambiguous, while this would be essential information for public health measures^5^. The whole genome of *T. mentagrophytes*, *T. interdigitale* and *T. indotineae* are very similar, and *T. mentagrophytes* and *T. interdigitale* can be distinguished with difficulty from each other using molecular characteristics^4,5^. *T. indotineae* is a newly identified dermatophyte species that is identical to genotype VIII within the *T. mentagrophytes*/*T. interdigitale* species complex, which was described by sequencing the Internal Transcribed Spacer (ITS) region of ribosomal DNA of the dermatophyte^3,4^. ITS provides the taxonomic basis for species identification in dermatophytes, and there are more than 10 ITS genotypes of *T. interdigitale*/*T. mentagrophytes* species complex can now be identified^6–17^. However, the nucleotide sequence of the translation elongation factor 1-α (*TEF-1α*) gene, which encodes a part of the protein translation machinery, was considered an alternative to rDNA and to have desirable properties for phylogenetic inference in other groups of pathogenic fungi^2,4,11,18^.

The established therapeutic approaches for treating dermatophytosis include the use of griseofulvin (GRI), as well as systemic or topical triazoles and allylamines such as itraconazole (ITZ) and terbinafine (TRB)^19^. Currently, terbinafine is the preferred choice due to its consistent clinical efficacy and lower recurrence rates^17,20^. However, there is a concerning increase in therapeutic failures, resulting in significant healthcare costs worldwide^5–11^. Additionally, there is a growing body of research on the resistance of dermatophyte species, specifically towards antifungal drugs like terbinafine^6,8–10,17,20–22^. The development of antifungal resistance in dermatophytes involves changes in genes responsible for producing enzymes targeted by antifungal agents or interfering with the interaction between antifungal agents and fungal enzymes. *T. indotineae* has become a problematic dermatophyte due to its predominantly in vitro genetic resistance to terbinafine owing to point mutations of the squalene epoxidase gene^3,6,10,14^. The multi-resistant *T. indotineae* has appeared to be spreading towards the world, with notable presence in Asian countries^5–10,14,21–24^.

The known molecular mechanism of resistance to TRB resistance is primarily based on specific point mutations in the *SQLE* target gene, resulting in a substitution of a single amino acid such as (Leu^393^Phe, Phe^397^Leu, Phe^415^Leu, His^440^, Ser^395^Pro)^22^. However, there seems to be an unpredictable relationship between the minimum inhibitory concentration (MIC) values and the clinical outcome in patients with dermatophyte infections^3,5–11,20–22^.

With respect to the rising cases of dermatophytosis and antifungal drug resistance in the clinical isolates in Iraq and the lack of valuable and accurate data related to this issue, this study aimed to evaluate clinical epidemiology of dermatophytosis and accurately identify the etiologic dermatophytes using ITS-rDNA and *TEF-1α* region sequences with a polyphasic approach. The antifungal drug susceptibility of isolated dermatophytes to TRB, ITZ, fluconazole (FLZ), and voriconazole (VCZ) was evaluated with the presence of point mutations in *SQLE* gene in TRB resistant strains, according to how these mutations affect the protein structure and function.

## Results

### Patient data and characteristics of isolated dermatophytes

**Figure 1:**
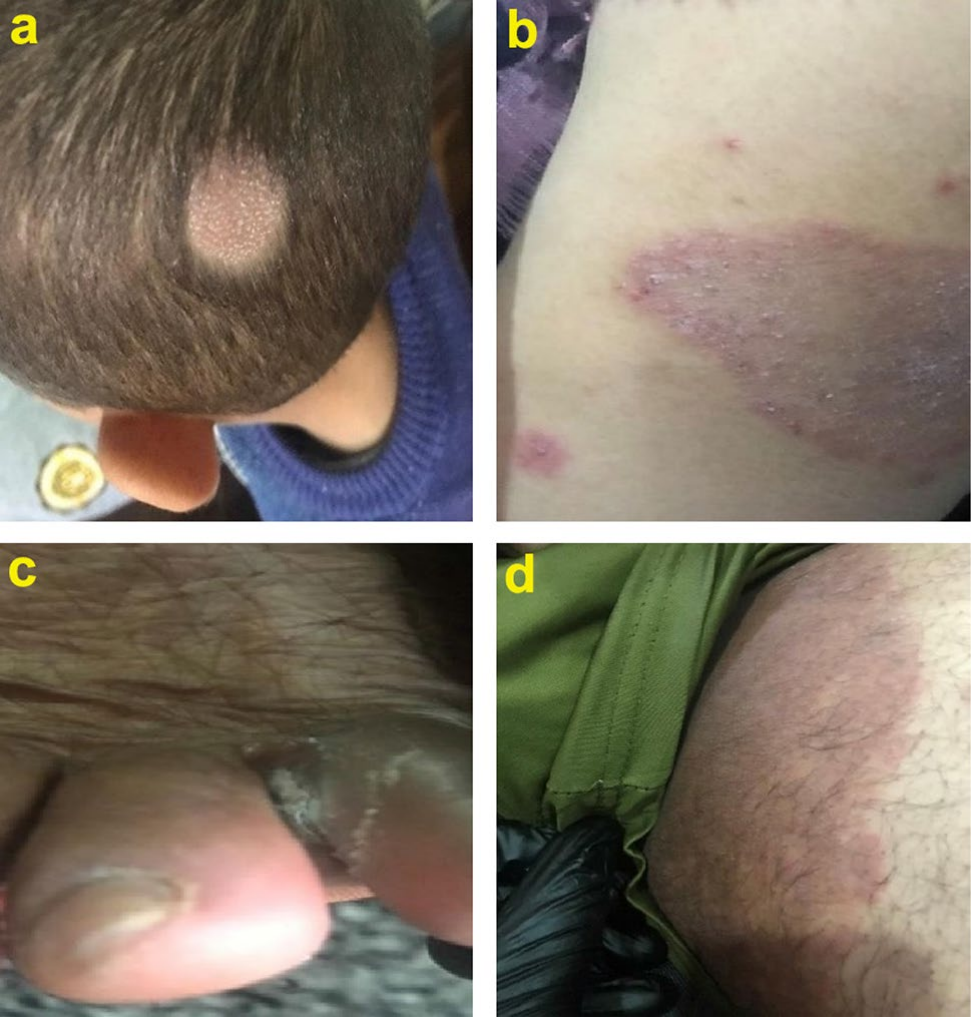
Clinical manifestations of dermatophytosis in patients referred to Al-Diwaneyah Teaching Hospital. Tinea capitis (**a**), tinea corporis (**b**), tinea pedis (**c**) and tinea cruris (**d**).

Table 1 illustrates the clinical data of 102 patients with proven dermatophytosis ranged from 5 to 60 years old, comprising 71 men (69.61%) and 31 women (30.39%). The most prevalent clinical manifestation was tinea corporis (37.25%) followed by tinea capitis (26.47%) and tinea cruris (18.63%). In contrast, tineae barbae and tinea manuum were the least prevalent clinical types (Fig. 1). *T. indotineae* topped the list of dermatophytes in terms of frequency, followed by *M. canis* and *T. mentagrophytes.*

**Table 1:** Distribution and prevalence of different types of dermatophytosis based on gender and age of patients.

Etiological agent	Clinical type	N (%)	Gender		Age					
			M	F	10 ≥	20 ≥	30 ≥	40 ≥	50 ≥	60 ≥
*T. indotineae* (n = 52)	Tinea corporis	18 (34.61)	12	6	3	7	2	4	2	0
	Tinea capitis	13 (25.0)	7	6	1	5	3	0	2	2
	Tinea cruris	12 (23.08)	12	0	2	2	4	2	0	2
	Tinea faciei	5 (9.62)	5	0	0	0	2	0	0	3
	Tinea barbae	1 (1.92)	1	0	0	0	0	1	0	0
	Tinea manuum	3 (5.77)	1	2	1	2	0	0	0	0
*T. mentagrophytes* (n = 20)	Tinea corporis	8 (40.0)	5	3	4	1	0	2	0	1
	Tinea capitis	5 (25.0)	2	3	1	0	1	2	1	0
	Tinea cruris	3 (15.0)	2	1	0	1	1	1	0	0
	Tinea faciei	2 (10.0)	1	1	0	0	2	0	0	0
	Tinea manuum	2 (10.0)	2	0	0	0	0	1	1	0
*M. canis* (n = 30)	Tinea corporis	12 (40.0)	7	5	2	4	2	1	2	1
	Tinea capitis	9 (8.82)	6	3	1	1	2	3	1	1
	Tinea cruris	4 (3.92)	4	0	0	1	2	0	0	1
	Tinea faciei	3 (3.88)	1	2	0	0	1	0	0	2
	Tinea manuum	2 (1.96)	2	0	0	2	0	0	0	0
Total (n = 102)	Tinea corporis	38 (37.25)	24	14	9	12	4	7	4	2
	Tinea capitis	27 (26.47)	15	12	3	6	5	5	4	3
	Tinea cruris	19 (18.63)	18	1	2	4	7	3	0	3
	Tinea faciei	10 (9.80)	7	3	0	0	5	0	0	5
	Tinea barbae	1 (0.98)	1	0	0	0	0	1	0	0
	Tinea manuum	7 (6.86)	5	2	1	4	0	1	1	0

Etiological agent and clinical type (p value < 0.98), clinical types and age (p value < 0.005), clinical type and gender (p value < 0.128).

### Molecular identification

**Figure 2:**
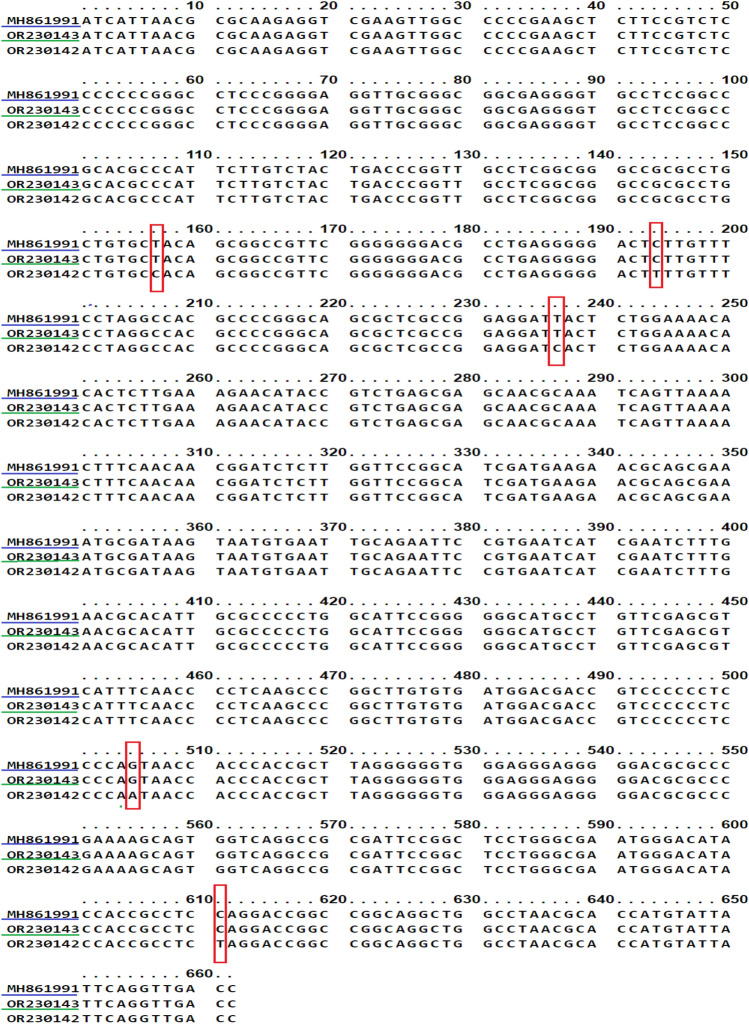
ClustalW multiple sequence alignment of *M. canis* isolates in the current study compared to CBS isolate from GenBank (blue underlined).* M. canis* type I green underlined and *M. canis* type II without underlined. The alignment created by Unipro UGENE 47.0 software. SNPS showed nucleotide substitution at positions T^157^C, C^194^T, T^237^C, G^505^A and T^611^C.

The ITS based method confirmed the presence of the following species: *T. indotineae* (accession no.; OR230110 to OR230119), *T. mentagrophytes* genotype XVII (accession no.; OR230121 to OR230126, OR398662 and, OR398663) and our identified two genotypes of *M. canis* (accession no.; OR230131 to OR230146)*,* where the frequency of dermatophytes was 52 (50.98%), 20 (19.61%) and 30 (29.41%), respectively.* T. indotineae* and *T. mentagrophytes* were identified as *T. interdigitale* (accession no.; OR256807 to OR256827) and *M. canis* (accession no.; OR256828 to OR256838) isolates were identified as one type when using *TEF-1α*. The obtained sequences were manually edited, blast analyzed, and deposited in GenBank (Table 2). The nucleotide variation of the two identified types of *M. canis* in comparison to the CBS496.86 strain (MH861991) was presented in Fig. 2.

**Table 2:** MIC values of four antifungal agents for 102 clinical isolates of *T. indotineae*, *T. mentagrophytes* and *M. canis* identified in the present study.

Antifungal drugs	MIC (μg/ml)	*T. indotineae* (n = 52)	*T. mentagrophytes* (n = 20)	*M. canis* (n = 30)
TRB	MIC range	0.003–4	0.003–4	0.007–0.125
	MIC_50_	0.06	0.155	0.03
	MIC_90_	0.25	1.3	0.125
	G-mean	0.046958005	0.103738991	0.031056213
	SD	0.803786706	1.195992743	0.037290041
VCZ	MIC range	0.01–1	0.03–4	0.01–0.125
	MIC_50_	0.125	0.125	0.06
	MIC_90_	0.475	0.625	0.125
	G-mean	0.117229239	0.125899645	0.052454883
	SD	0.254353997	1.200002714	0.035446252
ITZ	MIC range	0.01–1	0.01–4	0.01–8
	MIC_50_	0.125	0. 25	0.06
	MIC_90_	0.5	0.95	1.00
	G-mean	0.133957008	0.183028356	0.090704161
	SD	0.265900867	0.871140964	2.009837937
FLZ	MIC range	4–32	8–64	2–64
	MIC_50_	16	16	16
	MIC_90_	16	60.8	32
	G-mean	11.86489519	21.85664411	13.29980634
	SD	7.04735116	15.41564482	15.05606381

MIC: minimum inhibitory concentration; G-mean: geometric mean; SD: standard deviation.

### Phylogenetic analysis

**Figure 3:**
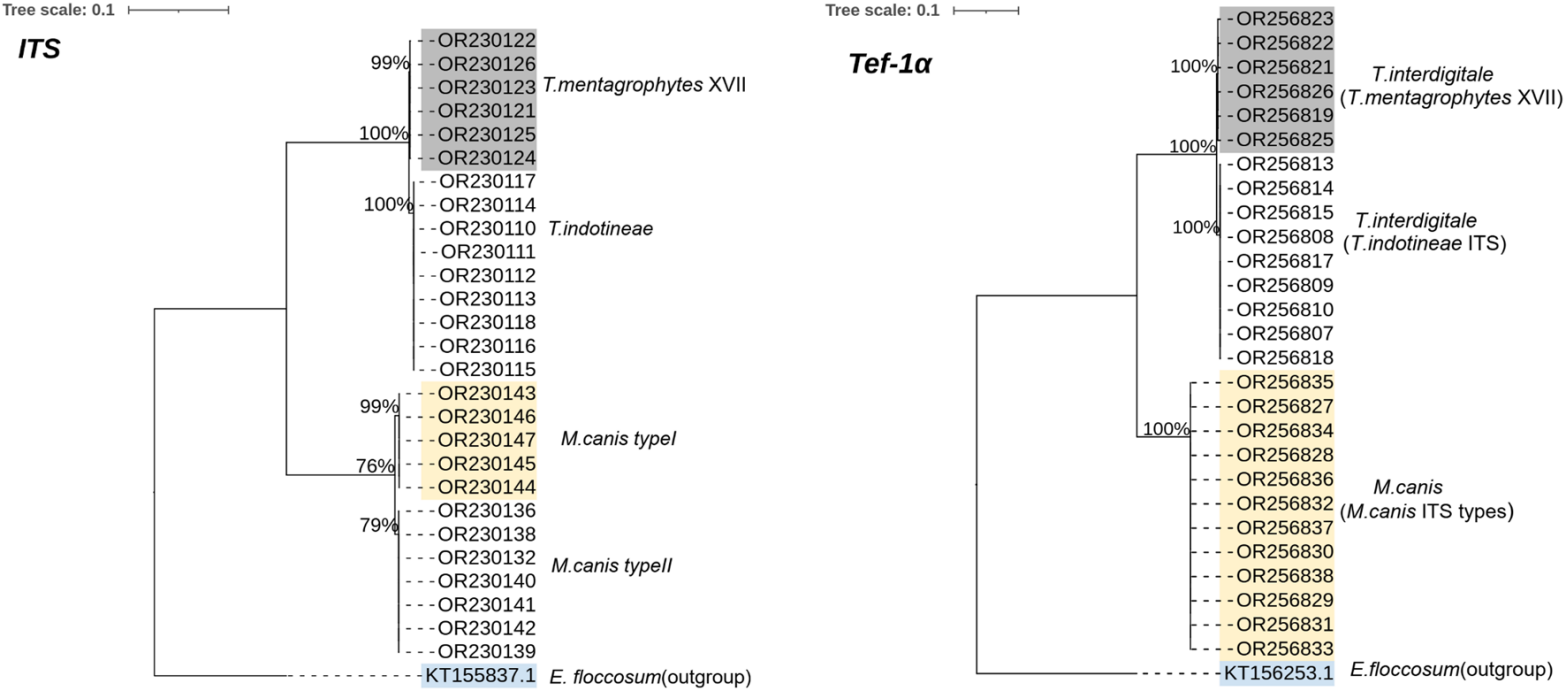
Maximum likelihood phylogenetic tree of dermatophytes included in the current study is based on ITS and *TEF-1α* sequences constructed by RAxML through CIPRES Science Gateway and edited by iTOL software. Values at the nodes indicate bootstrap percentages according to 1000 replicates, and branches with bootstrap values above 76% are shown (different clades are highlighted using different colors).

**Figure 4:**
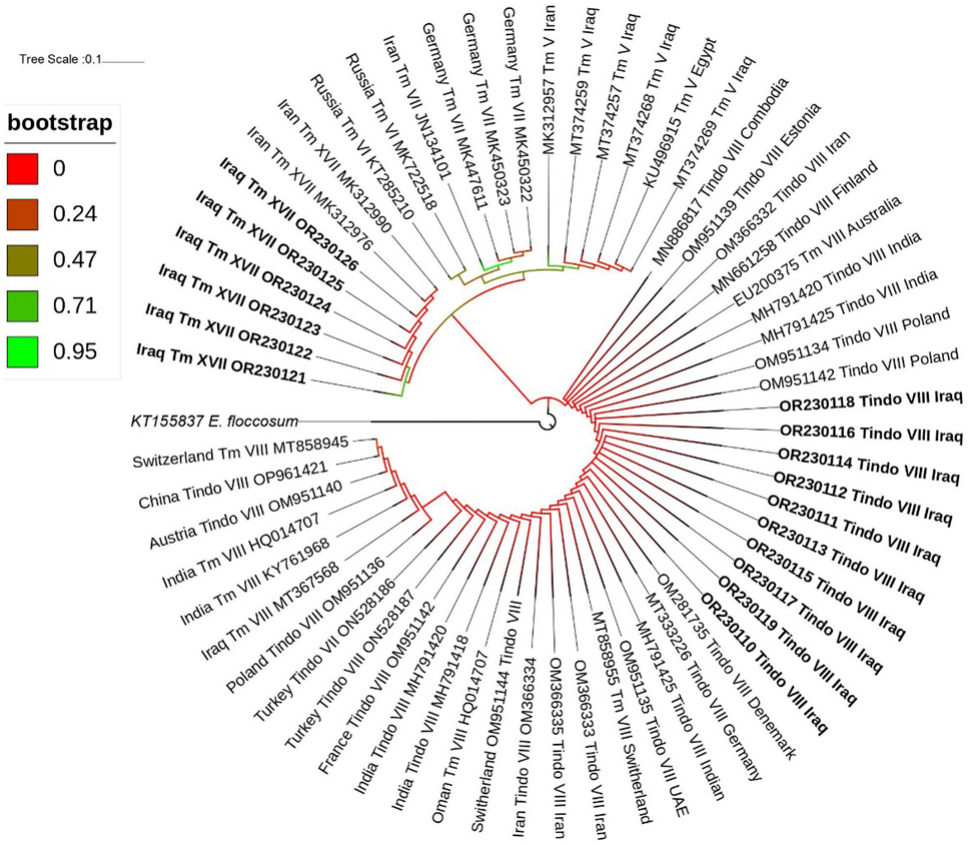
*T. mentagrophytes and T. indotineae*: Phylogenetic tree of Iraqi (designated by bold font) and international genotypes deposited in the GenBank database based on sequencing of the ITS* rDNA* region. Tree created through CIPRES Science Gateway and edited by iTOL software.

**Figure 5:**
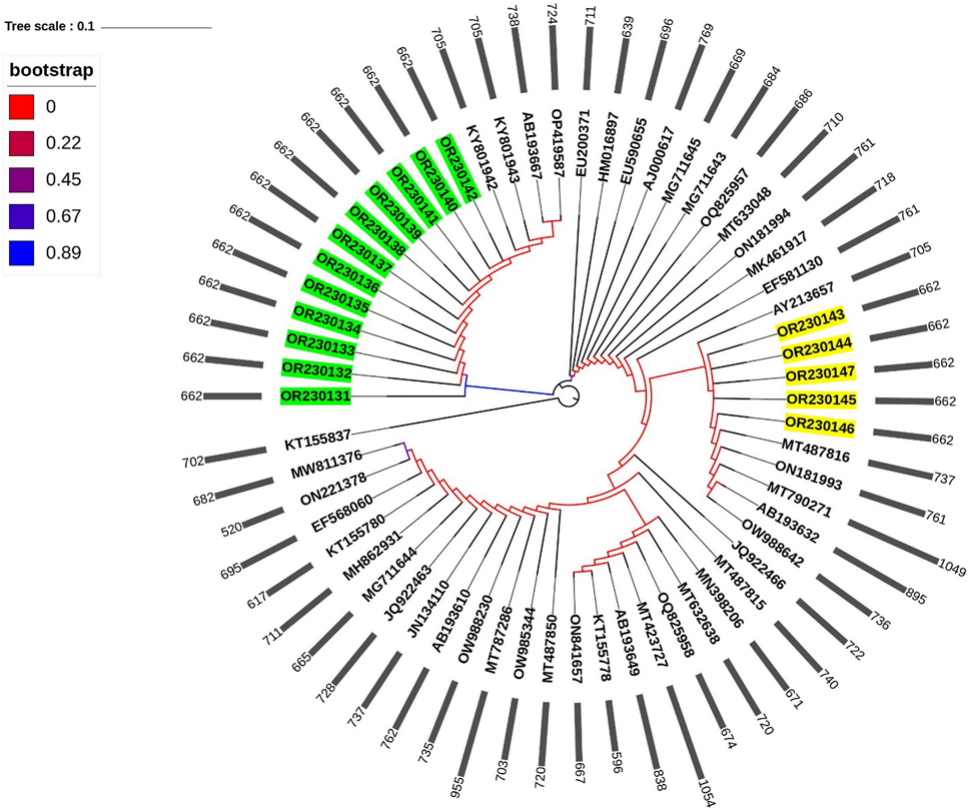
RAxML phylogenetic tree of *M. canis* of the present study and global strains: yellow background accession numbers refer to *M. canis* type I and green background accession numbers refer to *M. canis* type II. Simple bar annotations point to the sequence length (nts). The tree was created through CIPRES Science Gateway and edited by iTOL software.

RAxML was used to generate the phylogenetic tree for all identified species. Phylogenetic analysis categorized strains into four clades, forming separate clades (Fig. 3). *T. mentagrophytes* species clustered at the top of the tree, followed by *T. indotineae* while the basal position was occupied by the two types of* M. canis*. The *TEF-1α* gene-based phylogenetic tree showed three distinct clades, *T. indotineae* and *T. mentagrophytes* identified as *T. interdigitale* in separate clades and *M. canis* ITS types formed a cluster in one clade (Fig. 3b).* E. floccosum* (acession no.; KT155837) was considered the outgroup. Genotyping phylogenetic tree based on sequencing of the ITS rDNA showed *T. indotineae* (first report in Iraq) formed a cluster with all the international strains, whereas *T. mentagrophytes* XVII clustered with reported Iranian strains which can clearly be distinguished from all other genotypes V, VI and VII (Fig. 4). Identified *M. canis* type I was grouped with strains from USA (AY213657), Thailand (MT487816), Greece (ON181993), Cambodia (MT790271) Cambodia, Japan (AB193632) and Belgium (OW988642), while *M. canis* type II was closely related to Indian strains (KY801942, KY801943), Japan (AB193667) and Iraq (OP419587) as shown in Fig. 5.* E. floccosum* (accession no.; KT155837) acts as an outgroup (Fig. 5).

### Antifungal susceptibility testing

The antifungal susceptibility of 102 dermatophyte isolates to four antifungal drugs is shown in Table 2. TRB showed the highest efficacy compared to VCZ, ITZ, and FLZ. *T. indotineae* had the highest MIC_50_ for TRB (0.06 mg/L). *T. indotineae* and *T. mentagrophytes* had the same MIC value for VCZ (0.125 mg/L), while it was 0.06 mg/mL for *M. canis*. Among the tested antifungals, the highest MIC_50_ for ITZ (0.25 mg/L) was observed in* T. mentagrophytes*. Moreover, the dermatophytes displayed an equal MIC_50_ value for FLZ (16 mg/L). The highest geometric mean of antifungal drugs belonged to *T. mentagrophytes* for all drugs except TRB.

### Missense mutations of *SQLE*

**Figure 6:**
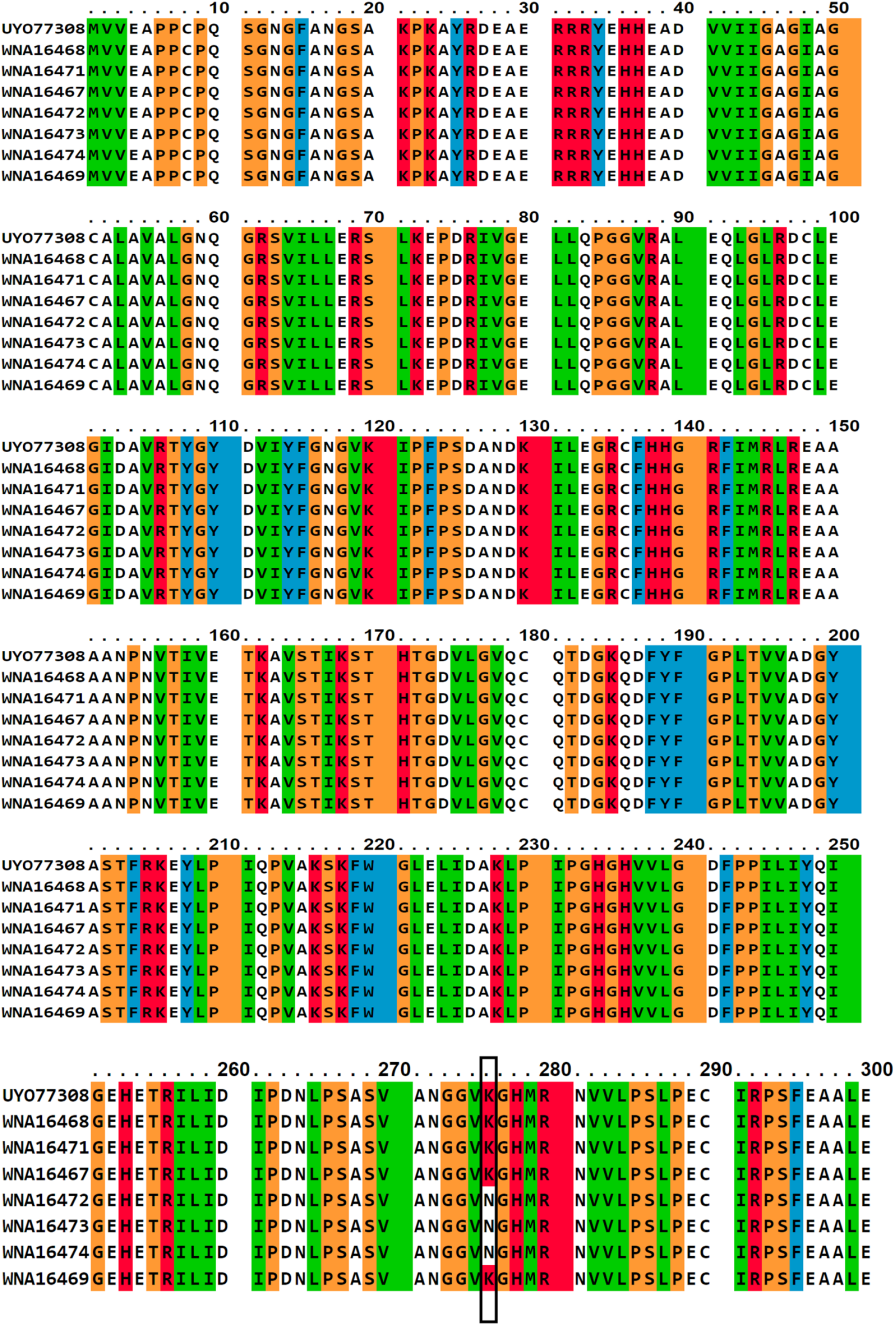

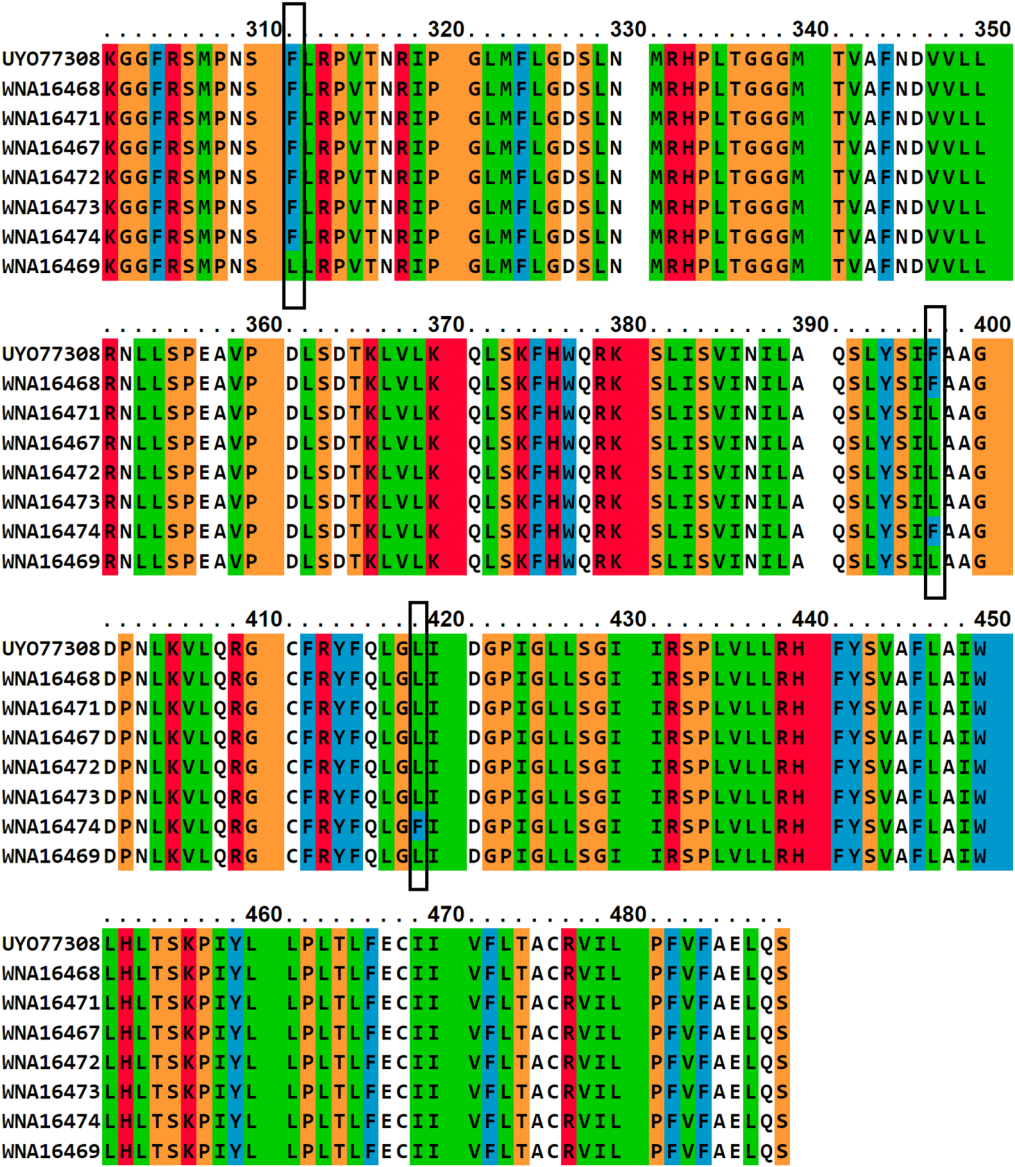
Amino acid substitutions of squalene epoxidase are marked by a black square. UYO77308 represents the wild type from GenBank, WNA16468 is the sensitive isolate (this study) and the rest of the isolates are terbinafine resistant. Multiple sequence alignment (MSA) of amino acids was performed by PSI-BLAST (http://www.ibi.vu.nl/programs/pralinewww/).

The mutations identified in the *SQLE* of TRB resistant strains identified in the present study are shown in Table 3. Point mutations were identified in three *T. indotineae* and two *T. mentagrophytes* genotype XVII strains. The mutation resulted in the replacement of phenylalanine with leucine Phe^397^Leu (T^1189^C) and Phe^311^Leu (T^931^C) in TRB resistant strain *T. indotineae* as illustrated in Fig. 6. Lys^276^Asn (G^828^C), Phe^397^Leu (T^1189^C), Leu^419^Phe (C^1255^T) were found in *T. mentagrophytes* genotype XVII.

**Table 3:** Mutations in the coding region of *SQLE* gene with amino acid substitutions.

Dermatophytes	ITS accession no	Nucleotide substitution	*SQLE* protein accession no.	Missense mutations in *SQLE*	MIC (mg/L)
*T. indotineae*	OR230111	T1189C	WNA16467	Phe^397^Leu	4
*T. indotineae*	OR230115	T1189C, T931C	WNA16469	Phe^397^Leu, Phe^311^Leu	4
*T. indotineae*	OR230117	T1189C	WNA16471	Phe^397^Leu	2
*T. mentagrophytes* XVII	OR230122	G828C, T1189C	WNA16472	Lys^276^Asn, Phe^397^Leu	4
*T. mentagrophytes* XVII	OR230124	G828C, T1189C	WNA16473	Lys^276^Asn, Phe^397^Leu	4
*T. indotineae*	OR230112	No substitution	WNA16468	No mutation	0.5
*T. indotineae*	OR230118	No substitution	WNA16470	No mutation	0.5
*T. mentagrophytes* XVII	OR230126	G828C, C1255T	WNA16474	Lys^276^Asn, Leu^419^Phe,	0.5

### 3D homology model of sqle and the effect of mutation

**Figure 7:**
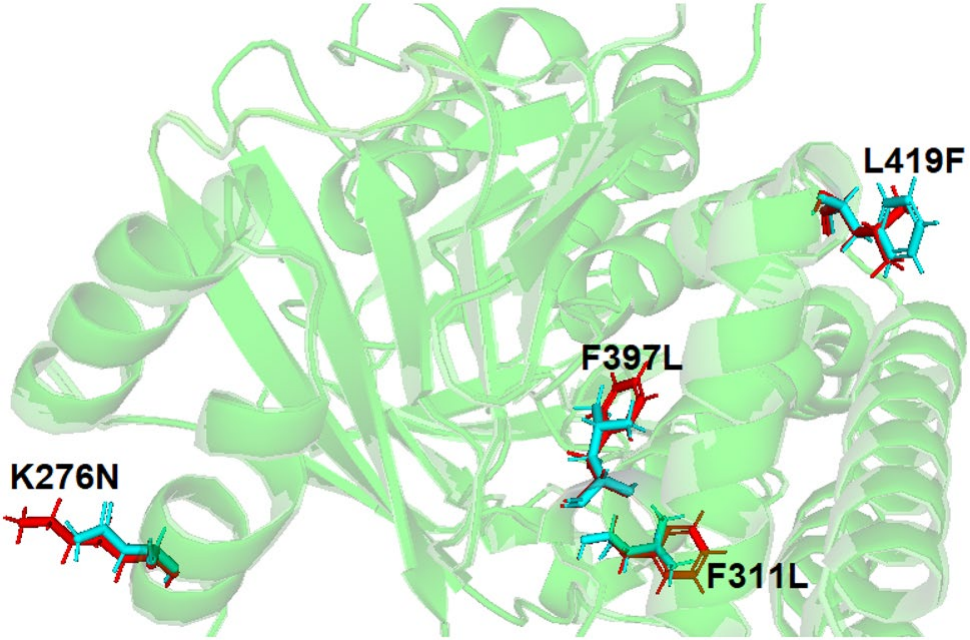
3D homology model of sqle mutant. Wild types are designated by a red color while mutant are designated by a blue color. Based on the ΔΔG results, the amino acid changes distort the protein structure. The model was predicted and assessed by SWISS-MODEL (https://swissmodel.expasy.org/) and visualized by Pymol software.

**Figure 8:**
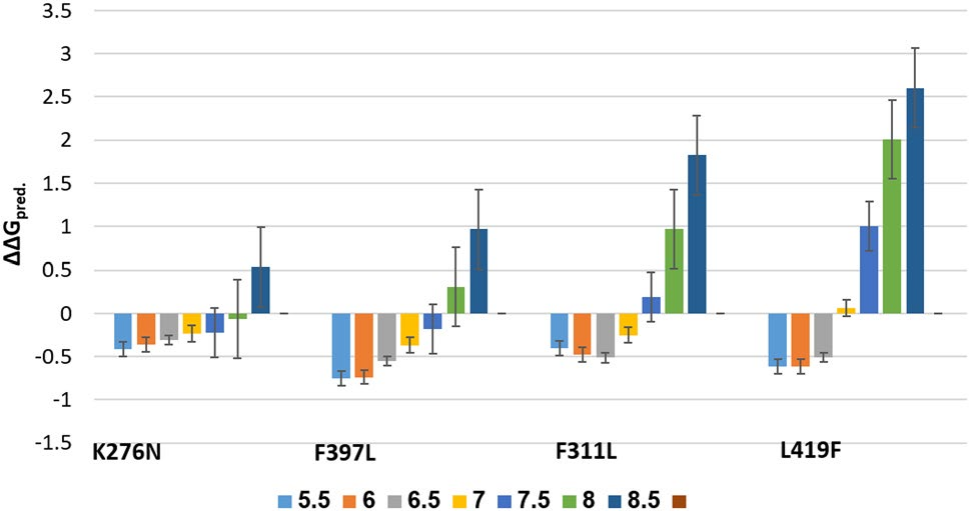
Analyzing point mutations in a range of pH (5.5–8.5) using the MAESTRO web server (https://pbwww.services.came.sbg.ac.at/maestro/web) shows that as the pH increases, the protein structure confirmation changes, and the protein stability deteriorates.

**Figure 9:**
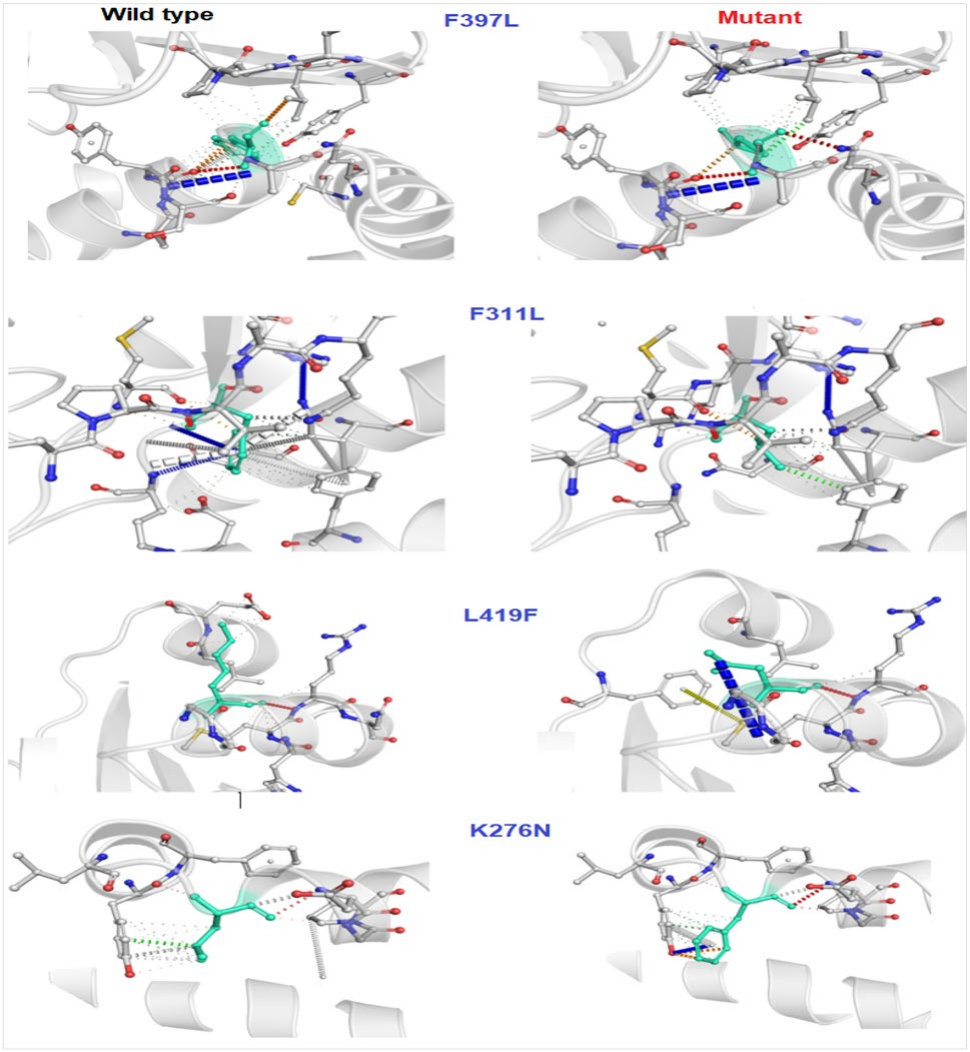
The figure illustrates the interatomic interaction between wild type and mutant, which shows bond disruptions in four residues by the Dynamut server (https://biosig.lab.uq.edu.au/dynamut/).

Results showed that the missense mutation led to a substitution of amino acids. Based on the homology modeling, the mutation in the binding site domain may lead to a failed drug enzyme interaction due to the smaller size of the mutant residue (Fig. 7). The results showed that Phe(F)^397^Lue(L) is a destabilizing mutation (ΔΔG < 0), while Lys^276^Asn, F^311^L and L^419^F are stabilizing mutations (ΔΔG < 0). Mutational analysis in a variation of pH (5.5–8.5) showed that increasing pH alters the protein structure confirmation, then the protein stability decreases (Fig. 8) and the interatomic interaction between wild type and mutant mutation residues also shows bond disruptions (Fig. 9).

## Discussion

Dermatophytes are the main cause of superficial mycoses in humans and animals. The main cause of increasing the incidence of dermatophytosis is not clearly understood but can vary depending on geographical location, migration patterns on different continents, changes in human lifestyles, and the medical approach to dermatophytosis in different health systems^24^. *T. mentagrophytes* species complex as an important causative of dermatophytosis has proven to be responsible for extensive infection, frequent relapses, and treatment failures^25^. The emergence and worldwide spread of terbinafine resistant *T. indotineae* (formerly named *T. mentagrophytes* genotype VIII) is a new challenge in the management of dermatophytosis^24–29^. Since very little has been documented about the prevalence and incidence of etiological agents of dermatophytosis especially *T. mentagrophytes* species complex in Iraq, we aimed to investigate the clinical epidemiology of dermatophytosis in suspected patients referred to Al-Diwaneyah and the antifungal susceptibility profiles of the identified etiologic species. Also, this study was aimed at providing additional insights into the genetic relatedness of the isolated strains and potentially shed light on their evolutionary relationships.

Our results demonstrated the emerging predominance of *T. indotineae* as the cause of tinea corporis cases. The most prevalent clinical manifestation was tinea corporis caused by all three identified species, including *T. indotineae*,* T. mentagrophytes* and* M. canis* followed by tinea capitis and tinea cruris. Tineae barbae and tinea manuum were the least prevalent types in our clinical cases. Also, males had twice as many clinical infections as females. These findings are consistent with previous reported research^20,25,27^. *T. indotineae* was reported as highly prevalent in both tinea corporis and tinea cruris^3,8,10,14,28,29^. Our results by ITS sequencing showed that the etiological agents of dermatophytosis in 102 proven cases were* T. indotineae* (50.98%), *T. mentagrophytes* (19.60%), and *M. canis* (29.41%). *T. indotineae* and *T. mentagrophytes* were identified as *T. interdigitale* and* M. canis* was identified as one type based on *TEF-1α* sequencing. Previous results showed consistency between the topologies of ITS and *TEF-1α* trees; however, the specificity and discriminatory power were higher with *TEF-1α* compared with ITS, which is particularly useful in some closely related species groups of dermatophytes^4,12,15,16^. *T. mentagrophytes* genotype complexes were distinguished on the basis of rDNA ITS and the partial *TEF-1α* gene including genotype VIII (segregated now as *T. indotineae*) in India which is characterized by elevated virulence and frequent occurrence of TRB resistance^7,12,13,17^. The single unique polymorphisms in anthropophilic and zoophilic* T.interdigitale* strains were revealed using ITS sequencing^15^. On the basis of the ITS data, Kano et al.^26^ described a new species, *T. indotineae*, for two strains. However, the whole genome of *T. mentagrophytes*, *T. interdigitale* and *T. indotineae* are very similar; Tang et al. reported that ITS and *TEF-1α* tree topologies were congruent, with a higher level of diversity in the ITS locus^4^. The combined *TEF-1α* and ITS marker sequences were used to establish the most robust phylogenetic relationships and integrated into the genotypic classification of dermatophytes^11–13,18^.

*T. indotineae* has also been reported to cause recalcitrant dermatophytosis in the world (e.g., Japan, China, Greece, Germany, France, North and Central America)^6,7,10,12,14,23,26–28^. In addition, it ranked as the second most frequent genotype among Iranian isolates, which is in line with our study’s findings^16,29^. The information of medical records for certain cases did not include any travel histories to India, Iran, or other countries. Also, it was the first report of *T. mentagrophytes* genotype XVII in isolated strains from Iraq that was previously reported in Iran (accession no; MK312976)^3,16^ however, Nenoff et al. reported *T. mentagrophytes* genotype V from two patients from Iraq^28^.

Antifungal resistant dermatophytosis outbreaks have been documented throughout India within the last decade, with some outbreaks reaching epidemic proportions^3,5,7,8^. The principal etiological agent is *T. mentagrophytes/T. interdigitale* and treatment failure occurs when resistance develops in these isolates^20^. Singh et al. reported TRB-resistant *T. mentagrophytes/interdigitale* complex isolated from tinea corporis/cruris exhibiting elevated MICs (4 to ≥ 32 μg/mL) to terbinafine and all harboring single-point mutations Leu^393^Phe or Phe^397^Leu in the *SQLE* gene^8^. Moreno-Sabater et al. found TRB-resistant *T. indotineae* isolates (MIC ≥ 2 μg/mL), harbored a Leu^393^Ser amino acid substitution, whereas *T. indotineae* TRB susceptible isolates were wild type or harbored the Ala^448^Thr amino acid substitution^9^. Taghipour et al., Salehi et al., and Pashootan reported *T. interdigitale* isolates were more sensitive to TRB than *T. mentagrophytes* and *T. indotineae* which is also consistent with our study^11,16,19,29^. Pashootan et al. found that *T. mentagrophytes/T. interdigitale* complex, genotype XXIX of *T. mentagrophytes* and genotype II of *T. interdigitale* displayed higher MIC_50_ values^29^. The authors suggested that certain genotypes within this species complex demonstrate a higher MIC in contrast to the others. In the current study, *T. indotineae* and *T. mentagrophytes* showed the highest resistance to antifungal drugs, while *M. canis* was the most susceptible species. *T. mentagrophytes* strains had the highest MIC_50_ for TRB and ITZ, in contrary to *M. canis* strains with the lowest MIC_50_. VCZ exhibited the most significant resistance effect against *T. mentagrophytes* genotype XVII. *T. indotineae* and *T. mentagrophytes* had the same MIC value for VCZ, while it was different for *M. canis*. All* T. indotineae*, *T. mentagrophytes* and *M. canis* strains displayed an equal MIC_50_ value for FLZ.

The most common causes of TRB resistance are point mutations that cause Leu^393^Phe and Phe^397^Leu amino acid substitutions in *SQLE* gene^6,8,9,11,22,29,30^. Shankarnarayan et al. reported the presence of the C^1191^A mutation among *T. mentagrophytes* complex isolates responsible for TRB resistance dermatophytosis in India^30^. In our study, the amino acid substitution mutation resulted in Phe^397^Leu (T^1189^C) and Phe^311^Leu (T^931^C) were found in *T. indotineae* and Lys^276^Asn (G^828^C), Phe^397^Leu (T^1189^C), and Leu^419^ Phe (C^1255^T) were found in TRB resistant *T. mentagrophytes* genotype XVII strains, respectively. TRB resistant *T. indotineae* harbored mutation Phe^397^L in *SQLE* gene has also been reported in many countries, which aligns with results of the current research^8,9,22,29,30^*. T. mentagrophytes* III* harbored missense mutations in *SQLE* gene corresponding to amino acid substitutions L^419^Phe and* T. mentagrophytes* VII harbored Lys^276^Asn^9^. Abastabar et al. also reported L^419^Phe substitutions in TRB resistant* T. mentagrophytes* strain, in accordance with our results which documented two mutations^31^. Phe^311^Leu was described for the first time in our *T. indotineae* isolate (*SQLE* accession no.WNA16469).

Analyzing how amino acid substitutions leads to drug resistance can provide insights into drug-enzyme interactions. Nowosielski et al. applied atomic 3D modeling to examine squalene epoxidase in *S. cerevisiae* isolates^32^. They figured out that the C-terminal region of the squalene epoxidase has the strongest drug-enzyme interaction at amino acids Phe^402^, Phe^420^, Phe^417^, Cys^416^, Val^92^, and Tyr^90^. Homology modeling has demonstrated that the Phe^397^Leu substitution results in structural destabilization of the enzyme in non-wild-type strains. This destabilization affects drug-enzyme binding, which agrees with the results of our study. MAESTROweb server results show that pH 8 and 8.5 had greater decreases in protein stability, especially in mutations F^397^ L and L^419^F^33^. The point mutation of all sites, which were described earlier in the results, visualizes disruption bonds like hydrogen bonds and hydrophobic interactions between different atoms of the protein. In interatomic interaction studies, the mutants have shown bond disruption, which has an effect on the structure and function of proteins.

## Conclusion

Due to the rising cases of dermatophytosis and antifungal drug resistance in Iraq, accurate identification of the causative species and the selection of effective antifungal drugs are necessary for successful treatments. Our study contributes to the growing body of knowledge on the geographic distribution and genotypic diversity of isolated dermatophytes, including *T. mentagrophytes*, *T. indotineae* and *M. canis* and highlights the utility of both ITS and *TEF-1α* region sequencing for accurate species identification in dermatophytes. ITS is more suitable for distinguishing between species, as it was capable of separating the two types of *M. canis*. According to our study, *T. indotineae* was the predominant organism causing superficial dermatophytosis outbreaks. Our findings revealed that substituting Phe^311^Leu for *SQLE* gene in *T. indotineae*, Phe^419^Leu and Phe^397^Leu in *T. mentagrophytes* XVII, could be used as a potential marker for the diagnosis of terbinafine resistant dermatophytes species regarding the selection of appropriate drugs for treatment. It is suggested that molecular typing using the high mobility group (HMG) transcription factor gene and other less frequent mutations in TRB resiatant dermatophytes be investigated with wide distribution studies in dermatophytosis in different parts of Iraq.

## Material and methods

### Patient samples

This study included 102 dermatophyte isolates collected from patients who visited Al-Diwaneyah Teaching Hospital, Department of Dermatology between 2021 and 2022. All patients (a parent and/or legal guardian regarding children) signed a consent form after being informed of the objectives of the study, and the confidentiality of participants’ personal information was protected as rights to refuse to take part in the study as well as to withdraw at any time during the study period were given. All the information obtained from the study patients was coded to maintain confidentiality. When the participants were found positive for dermatophytosis, they were reported to the hospital, and the clinician treated them accordingly. All methods were performed following the relevant guidelines and regulations.

### Identification of isolated dermatophytes

The skin, hair, and nail samples were collected based on a standard practical guide for the diagnosis of fungal infections. The collected samples were subjected to direct microscopic examination using 20% KOH. The microscopic fungal positive samples were aseptically inoculated on mycobiotic agar (Merck, Germany) plates and incubated at 25 °C. The plates were periodically examined for the growth of dermatophytes every other day for 4 weeks. The initial identification of dermatophytes was carried out through morphological microscopic and culture characteristics. Additionally, five standard strains including *T. interdigitale* PTCC 5054, *M. canis* PTCC 5069, *N. gypsea* PTCC 130396, *T. verrucosum* PTCC 10694, and *T. rubrum* PTCC 5808, were prepared from the Pathogenic Fungi culture Collection of the Pasteur Institute of Iran (http://fa1.pasteur.ac.ir/pages.aspx?id=1152).

### Molecular identification

To confirm the identification of dermatophytes, all fungal isolates were cultured on mycobiotic agar (Merck, Germany) and incubated at 28 °C for 7 days. DNA of the isolates was extracted using a genomic DNA extraction kit (ExoGene, Iran) according to the manufacturer’s instructions by adding 200 mg of glass beads (0.2 mm diameter). The extracted DNA was dissolved in 50 μl of Tris/EDTA (TE) buffer, and stored at − 20 °C untill it was used.

The ITS region was amplified using primers ITS1 (5ʹ-TCCGTAGGTGAACCTGCGG-3ʹ) and ITS4 (5ʹ-TCCTCCGCTTATTGATATGC-3ʹ)^11^. The *TEF-1α* region was amplified using primers forward (5ʹ-CACATTAACTTGGTCGTTATCG-3ʹ) and reverse (5ʹ-CATCCTTGGAGATACCAGC-3ʹ)^18^. Each mixture contained 12.5 μl of 2× master mix (Ampliqon, Denmark), 1 μl of DNA template, 0.3 μM of each primer, and enough distilled water to reach a final reaction volume of 25 μl. Negative controls (water instead of fungal DNA) were added to each PCR. The reaction mixture was initially denatured at 95 °C for 5 min followed by 35 cycles of 30 s at 94 °C, 30 s at 58 °C, and 45 s at 72 °C, and a terminal extension step of 72 °C for 5 min. Subsequently, PCR products were electrophoresed on a 1% agarose gel in TE buffer^11^.

To carry out the sequencing, PCR products were processed using ABI PRISM BigDye Terminator Cycle Sequencing Ready Reaction Kit from Applied Biosystems (CA, USA). To identify and compare fungi, two web databases, specifically CBS and BLASTn were applied. With Unipro UGENE 47.0 software, the sequences of isolates were manually edited and subjected to ClustalW pairwise alignment and then the sequences were deposited in GenBank (Table 2). *T. interdigitale/T. mentagrophytes* species complex has been determined through ITS genotyping based on the studies by Heidemann et al.^15^ and Taghipour et al.^16^.

### Phylogenetic analysis

For phylogenetic tree, the sequences were analyzed using RAxML (https://www.phylo.org) version 8.2^34^ running on the CIPRES Science Gateway^35^. In RAxML, optimization was performed using the GTRCAT option. For maximum likelihood, the bootstrap values were 1,000 replicates with one search replicate per bootstrap replicate. *E. floccosum* was used as an outgroup. iTOL (https://itol.embl.de) is an online tool used for the display, annotation, and management of phylogenetic trees.

### Antifungal drug susceptibility

The fungal spore count was adjusted to 3 × 10^3^ CFU/ml in RPMI-1640 medium from 102 isolated dermatophytes. Antifungal drug susceptibility assay was performed based on CLSI document M38-3rd broth microdilution method^36^. The final concentrations of terbinafine (4–0.003 µg/ml), voriconazole (16–0.01 µg/ml), itraconazole (16–0.01 µg/ml) and fluconazole (64–0.06 µg/ml) (Sigma-Aldrich, St. Louis, USA) were prepared using RPMI-1640 medium and MOPS buffer (Sigma-Aldrich, USA) including 0.2% glucose and phenol red, without bicarbonate, with a final pH of 6.9–7.1. Fungal spores were inoculated with a twofold serial dilution of antifungal drugs in 96-well cell culture plates and incubated at 30 °C for 4 days. The minimum inhibitory concentration (MIC) range, geometric mean, MIC_50_, and MIC_90_ were calculated based on duplicate tests. MIC was determined to be approximately 80% or more reduction in growth compared with the control well. *T. rubrum* (PTCC 5143) and *Candida parapsilosis* (ATCC 22019) were used as quality controls^21^.

### Detection of *SQLE* missense mutations in terbinafine resistant strains

The *SQLE* gene mutations in TRB resistant strains were investigated using the primers TrSQLE-F (5ʹ-ATGGTTGTAGAGGCTCCTCCC-3ʹ) and TrSQLE-R (5ʹ-CTAGCTTTGAAGTTCGGCAAA-3ʹ)^22^. PCR reactions were achieved following the protocol mentioned by^21^. PCR products from the *SQLE* region were sequenced using the ABI PRISM BigDye Terminator Cycle Sequencing Ready Reaction Kit. BioEdit 7.2 software was used to edit and optimize the sequences to deposit in GenBank. To visualize the presence of mutations, PSI-BLAST (http://www.ibi.vu.nl/programs/pralinewww/) was used for this purpose. The wild type (UYO77308) from GenBank and the sensitive isolate (WNA16468) of the current study were used to find out the amino acid substitutions in TRB resistant isolates.

### Three-dimensional (3D) homology model and the effect of mutation in *SQLE*

The SWISS-MODEL server (https://swissmodel.expasy.org) was used to predict the protein's 3D structure of *SQLE*, only the highest sequence identity scores were deemed significant. The assessment and quality of protein 3D structure was performed using SWISS-MODEL tools. By calculating ΔΔG (Gibbs free energy), the effect of a point mutation on protein structural stability could be determined. To accomplish this goal, the ERIS server was used, permitting the induction of point mutations, and providing the calculation of the ΔΔG. The value of ΔΔG > 0 refers to a destabilizing mutation and vice versa. PyMOL, a widely used molecular graphics software, was used for protein visualization. Structure-based point mutation analysis with various pH (5.5, 6, 6.5, 7, 7.5, 8, and 8.5) was done by the MAESTROweb server (https://biwww.che.sbg.ac.at/maestro/web). A negative value of ΔΔG means an increase in the destability of the protein, whereas a positive value depicts a diminishment in the stability of the protein^33^.

The essential studies of point mutation are the analysis of interatomic changes from a wild type to a mutant, which was done by the DynaMut web server, which allows the visualization of all interactions calculated by the Arpeggio web server (http://biosig.unimelb.edu.au/dynamut/).

### Statistical analysis

Quantitative data of the MIC range, geometric mean MIC, MIC_50_, and MIC_90_ were subjected to statistical analysis using one-way ANOVA and multiple comparisons tests using the statistical SPSS package version 19. P values of < 0.05 were considered significant.

### Ethics declarations

This project was found to be according to the ethical principles and the national norms and standards for conducting Medical Research in Iran and has been approved by the research ethics committee Tarbiat Modares University with code IR.MODARES.REC.1399.013.

### Informed consent

Informed consent to participate and publish was obtained from all the participant as mentioned in the part of patients samples.

### Supplementary Information

Supplementary Table S1.

## Data Availability

ITS-rDNA and *TEF-1α* accession numbers of dermatophytes and *SQLE* gene sequences in terbinafine resistant strains are deposited in GenBank (included in supplementary Tables S1 and 3). The datasets generated during and/or analyzed during the current study are available from the corresponding author on reasonable request.
