# Supplementary material for: Epidemiological trends, antifungal drug susceptibility and SQLE point mutations in etiologic species of human dermatophytosis in Al-Diwaneyah, Iraq
Source: Sci Rep. 2024 Jun 3;14:12669. doi: 10.1038/s41598-024-63425-w (PMC11148054; doi:10.1038/s41598-024-63425-w)
Supplement: Supplementary file 1 — Supplementary Table S1. [file 41598_2024_63425_MOESM1_ESM.docx]

**Table S1.** ITS-rDNA and *TEF-1α* GenBank accession numbers of dermatophytes included in this study

| **GenBank accession Number** | **Dermatophyte** | **DNA region** | |
| --- | --- | --- | --- |
| OR230110, OR230111, OR230112, OR230113 OR230114, OR230115 OR230116, OR230117 OR230118, OR230119 | *T. indotineae* (type VIII) | | ITS-rDNA |
| OR230121, OR230122, OR230123, OR230124 OR230125, OR230126, OR398662, OR398663 | *T. mentagrophytes* (type XVII) | |  |
| OR230131, OR230132, OR230133 OR230134 OR230135, OR230136 OR230137, OR230138 OR230139, OR230140, OR230141, OR230142 OR230143, OR230144, OR230145, OR230146 | *M. canis* (Arthroderma otae) | |  |
| OR256807, OR256808, OR256809, OR256810 OR256811, OR256812, OR256813, OR256814 OR256815, OR256816, OR256817, OR256818 OR256819, OR256820, OR256821, OR256822 OR256823, OR256824, OR256825, OR256826 OR256827 | *T. interdigitale* | | *TEF-1α* |
| OR256828, OR256829, OR256830, OR256831 OR256832, OR256833, OR256834, OR256835 OR256836, OR256837, OR25683 | *M. canis* | |  |
